# Societies at risk: the correlation between intensity of armed conflict and child health during the civil war in South Sudan

**DOI:** 10.1186/s13690-025-01523-5

**Published:** 2025-02-20

**Authors:** Caroline de Groot, MHD Bahaa Aldin Alhaffar, Anneli Eriksson

**Affiliations:** Karolinska Institutet, Stockholm, Sweden

**Keywords:** South Sudan, Armed conflict, Child health, Global acute malnutrition, Under-five crude mortality, Panel data analysis

## Abstract

**Background:**

Armed conflict severely impacts children's health, leading to malnutrition and increased child mortality. The republic of South Sudan gained independence from Sudan in 2011 and suffered from seven-years civil war between December 2013–2021. The war led to mass population displacement both internally and externally and worsened the health status of the population, especially the children.

**Aim:**

To investigate the effect of conflict intensity on global acute malnutrition and under-five crude mortality rate in South Sudan during the civil war between 2014–2021.

**Methods:**

The study used an ecological panel data analysis of armed conflict data from Uppsala Conflict Data Program (UCDP) and child health data from Standardized Monitoring and Assessment of Relief and Transitions surveys conducted in 2014–2021. Child health is evaluated as global acute malnutrition (GAM) and under-five crude mortality rate (U5CMR). The study analyzed the correlation between the intensity of conflict and the prevalence of malnutrition and under-five crude mortality on a state level. Internal displacement and food prices were used as indirect effects of conflict. One year lag effect regressions were used to estimate potential correlation between child health and armed conflict.

**Results:**

Total number of conflict related deaths between 2014–2021 was on best estimate (9,577), and on high estimate (13,178). The average GAM rate for the same period was (15.29%), and U5CMR was (0.77). Data analysis showed a significant correlation between the high estimate of conflict intensity with GAM (.047), and with U5CMR (.043). Internal displacement and food prices had a significant correlation with GAM (*P* = .048, *P* = .016), but no significant correlation was noticed with U5CMR. Best estimate of conflict intensity did not show a significant effect on children health variables.

**Conclusion:**

The effect of conflict on children’s health outcome is complex and multifactorial. The high estimate of conflict intensity from UCDP showed significant correlation with the health outcome, while best estimate did not have significant correlation, this could be due to limited child health data, underreporting of conflict-related deaths, and a small sample size. The study suggests that other factors such as food prices and displacement might play an additional factor that increases the effect of conflict intensity on child health outcomes. The study underscores the challenge of data scarcity in researching health determinants in South Sudan.

**Table:** 

Text box 1. Contributions to literature
• Addresses the gap in research on South Sudan, an underserved country with limited health literature.
• Explores the impact of context-specific determinants of health in a conflict-affected environment.
• Investigates how armed conflict specifically affects child health outcomes.
• Examines various mechanisms and hypotheses that connect armed conflict to child health issues.

## Introduction

Armed conflicts remain a pervasive and devastating phenomenon, significantly impacting the health and well-being of populations and contribute substantially to the global burden of disease, causing deaths and injuries [1]. Despite the aim set by the Sustainable Development Goals, to mitigate the frequency and intensity of these conflicts [2], the prevalence of conflict continues to rise globally [3]. Children are profoundly affected by the physical and psychological impacts of violence, including displacement and deprivation. Over half of the global child population resides in conflict-affected countries [4], with approximately 450 million children living in active conflict zones in 2021. This scenario underscores the direct and indirect effects of armed conflict on children’s health, ranging from death and injury to displacement and food insecurity [4].

### Theoretical framework

**Fig. 1:**
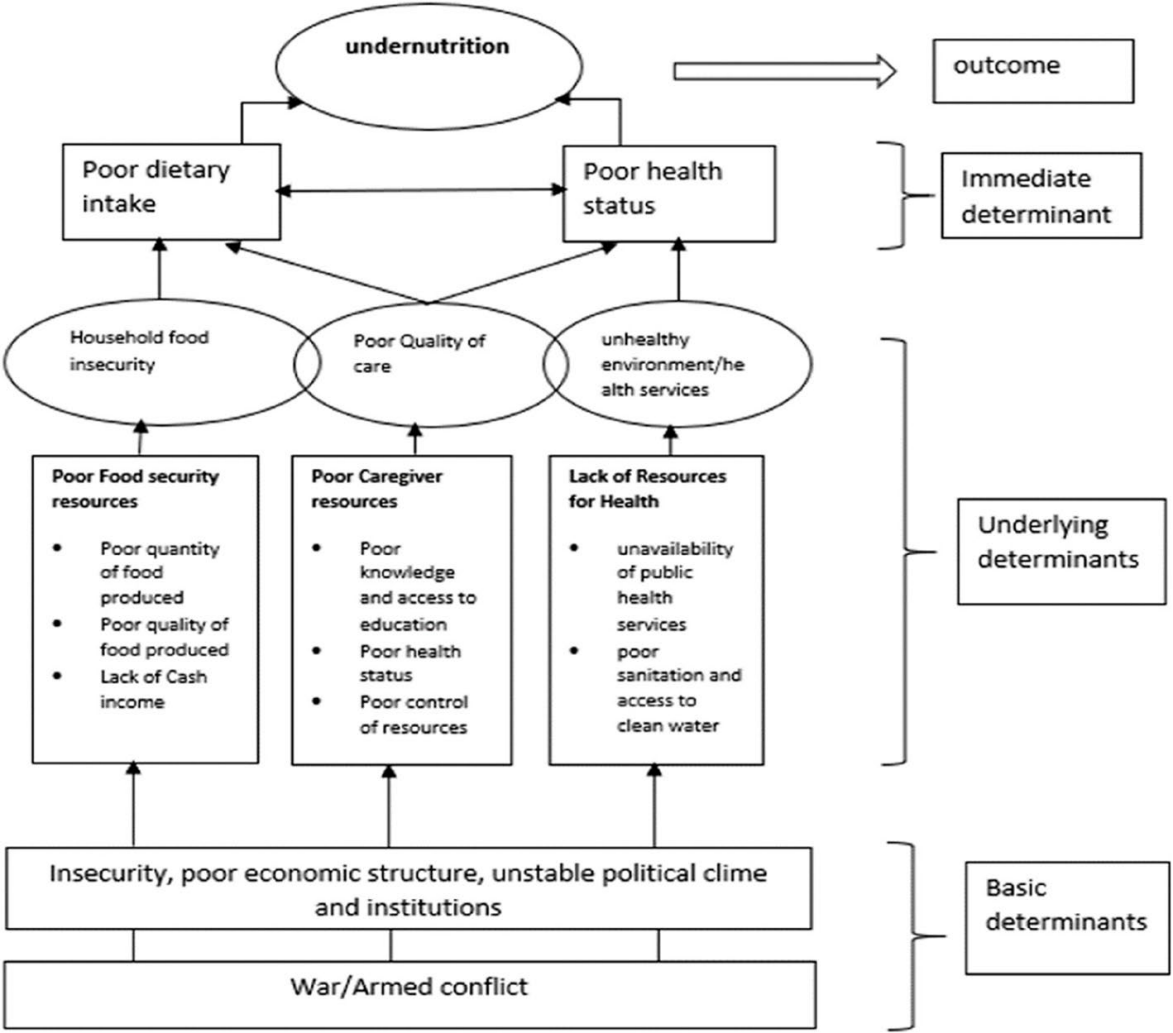
UNICEF conceptual framework on the effect of conflict on malnutrition as adopted by Makinde et.al 2023 [5]

The United Nations Children’s Fund (UNICEF) conceptual framework elucidates the complex impact of conflict on child nutrition, mapping the progression from basic determinants like war, insecurity, and weak economic conditions to underlying factors such as food insecurity and insufficient caregiver resources, culminating in child malnutrition (Fig. 1). This framework highlights how conflict-induced disruptions in food security, healthcare access, and environmental health drive malnutrition among children under five, emphasizing the critical role of accessibility to resources and services. By impairing access to essential needs like stable agricultural environments, sufficient food, safe water, and healthcare, conflict significantly elevates the risk of undernutrition and mortality in affected populations [5, 6].

The Republic of South Sudan provides a striking example of these effects. Emerging from a seven-year civil war, the country faces a fragile health system and low rankings in health and development indices [7, 8]. South Sudan’s history is marked by prolonged conflicts, including a two-decade-long civil war prior to its independence in 2011 [9, 10]. Post-independence, the country experienced another civil war from December 2013 to 2021, with significant political and ethnic divisions contributing to the conflict [11, 12]. The lack of reliable data obscures the true extent of casualties, but estimates suggest as many as 190,000 deaths during this period [13], while other sources give lower estimates [14]. South Sudan has some of the world’s worst health indicators, as the war have left a significant portion of the population in extreme poverty, and reliant on humanitarian aid [15], with South Sudan ranking last in the Human Development Index (rank 191 out of 191) [16], with high maternal and under-five mortality rates [17, 18].

This research investigates the repercussions of armed conflicts on child health within the context of South Sudan. Prior investigations have linked conflict to a variety of health outcomes but have not concentrated on South Sudan which has a complicated context [19–23]. Our study aims to address this gap by examining how the intensity of armed conflict influences child health in South Sudan throughout its civil war from 2013 to 2021. It specifically investigates the impact on global acute malnutrition and the crude mortality rate among children under five on a state level, offering insights into the direct health challenges faced by children in conflict-affected areas.

#### Aim of the research

To investigate the effect of conflict intensity on global acute malnutrition and under-five crude mortality rate in South Sudan during the civil war between 2014–2021.

## Methods and materials

### Study design and settings

The study utilized an ecological panel data analysis approach to investigate the link between armed conflict and child health indicators [24]. The study focused on the period of the South Sudan's civil war from 2014 to 2021. State-year was the primary unit for analysis, with the child health indicators of global acute malnutrition rate and under-five crude mortality rate at the forefront. The study population was children under the age of five across the ten states of the republic of South Sudan [24].

#### Epidemiological variables

**Fig. 2:**
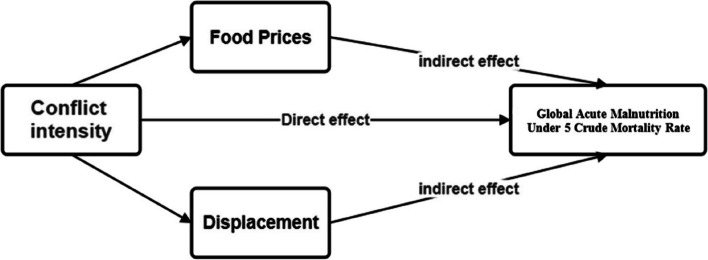
Hypothetical framework for the effect of conflict on children health

- Conflict-related deaths: Conflict intensity can be assessed through various variables, including economic, social, and public health impacts [25, 26]. However, this study adopted the Uppsala Conflict Data Program (UCDP) approach, focusing on the quantifiable metric of conflict-related death [9]. Data from UCDP were classified as a low estimate, best estimate, and high estimate of number of conflict-related deaths. The low estimate consists of the aggregated low estimates for all incidents. The best estimate represents the most accurate number based on the most reliable sources, meanwhile the high estimate captures the highest reported fatalities, including uncertain cases or less reliable Figures [9].
- Displacement and food prices: The indirect effects of conflict were studied through displacement and food prices, both of which are consequences of conflict that affect the nutritional outcome of the children [27]. Displacement figures were derived from the number of individuals internally displaced due to conflict, indicating the social and economic upheaval that exacerbates the public health crisis [28]. While food prices variables focused on white sorghum because of its importance in the South Sudanese diet, making up to 76% of the food supply. As the main source of nutrition in the country, the price of sorghum serves as an important indicator of food accessibility and, by extension, nutritional security [29].
- Child health variables: Additionally, we examined Global Acute Malnutrition (GAM), defined as the prevalence of severe or moderate acute malnutrition among children aged 6–59 months. GAM rate exceeding 15% in this age group indicates a nutrition crisis, highlighting the dire circumstances faced by the youngest and most vulnerable. The under-five crude mortality rate (U5CMR) was another critical measure, representing the rate of deaths among children under five years within a specified period and population, U5CMR > 2/10000/day indicate a severe crisis and a significant public health emergency that requires an immediate response [30]. Figure 2 illustrates the hypothetical framework of this study.

### Data collection and source

The study included data covering the armed conflict direct effect (conflict intensity), armed conflict indirect effect (food prices, population displacement), and child health data covering global acute malnutrition and under-five crude mortality rate. The sources of the data included in the study were as follow:

- Armed conflict data was sourced from the UCDP, both best and high estimate of conflict related death was used [9]. Only data that specify the location (per state), year (from 2014–2021), and number of deaths are used. Conflict intensity is presented in the study as the case count of conflict related death (best and high estimate), and the death rate which is calculated as the number of conflict-related deaths per 100,000 inhabitants, based on 2020 OCHA population [15].
- Child health data is obtained from Standardized Monitoring and Assessment of Relief and Transitions (SMART) surveys. We obtained an extract of the database consisting of the results of 313 surveys conducted by 52 different organizations during the eight years of conflict in the ten subnational nutrition clusters of South Sudan. 311 out of 313 surveys were included in the analysis. Two SMART surveys (year 2015) from Abyei region in northern South Sudan are excluded due to conflict with Sudan and the undefined border area. SMART surveys were conducted across all regions, with Jonglei and Upper Nile having the highest number of surveys, while Central and Western Equatoria recorded the fewest. The year with the highest number of missing values was 2020, with only five surveys conducted. This trend continued into 2021, largely attributed to the global impact of the COVID-19 pandemic, which disrupted public health surveillance and data collection efforts in many countries worldwide. To fill the missing GAM Rates from 2020, 78 GAM rates were included in the study as they were extracted from the Humanitarian Data Exchange platform (HDX) for the year 2020, also managed from OCHA [31, 32]. The data was collected by the Nutrition Clusters and included the state, county, year, and nutrition GAM rates. Global acute malnutrition rates were measured as percentage for state per year and under-five crude mortality rate which was calculated using this formula: [30]

$$\text{U}5\text{CMR }= (\text{total number of }<5\text{ death}/\text{total number of }<5\text{ children }*\text{ number of days in the period studied}) * 10.000\text{ persons }=\text{ Deaths per }10.000\text{ children under }5\text{ per day}$$

U5CMR was presented as death per 10,000 children/day, from 2014 to 2021.

- Food prices: Data regarding food prices in South Sudan was obtained from World Food Program (WFP) [33]. The item was white sorghum which is priced in 3.5 kg unit, and the prices was in USD for comparison with other studies [34, 35].
- Displacement: Data regarding internal displacement in South Sudan was obtained from the internal displacement monitoring center (IDMC), this database has the number of internally displaced population per year and state [28].

### Statistical analysis

The statistical analysis involved descriptive statistics to provide insights into the trends and magnitudes of armed conflict and child health issues. Moreover, we conducted panel data analysis utilizing logistic regression models, incorporating a one-year lag of the data to account for temporal dynamics to explore the potential influence of armed conflict on child health outcomes, and Pearson’s correlation coefficient to assess the strength, direction of associations, and the significance of the results. We choose a one-year lag as data was analyzed as annual averages. A one-year lag helps capture delayed or long-term impacts of armed conflict on child health, as many consequences of armed conflict (e.g., disruptions in healthcare, food insecurity, psychological trauma) may not manifest immediately. By lagging the data, we aimed to account for temporal dynamics and better estimate how conflict affects health over time. A three-months' time lag may have captured more immediate consequences but will not capture delayed effects like the cumulative impacts of sustained conflict. Using a time lag longer than one year could introduce several confounding factors, therefore, we chose one year as optimal. SPSS V.22 was used to perform the analysis of the data, and the results were illustrated through marginal effect plots.

## Results

### Descriptive analysis of the results

From 2014 to 2021, the total number of conflict-related deaths estimated by UCDP was 9577 for the best estimate, and 13178 for the high estimate conflict intensity. The highest number of deaths was in 2014 (2480 and 3940 for best and high estimate respectively) and decreased during the years to the lowest point in 2021 (891 and 930 respectively), see Table 1.

**Table 1:** Descriptive analysis of conflict intensity, child health, food prices and displacement in South Sudan (2014–2021)

		**CIBE**^**a**^	**CIHE**^**b**^	**GAM**^**c**^	**U5CMR**^**d**^	**Food prices**^**e**^	**Internal Displacement **^**f**^
		**Sum**	**Sum**	**Mean**	**Mean**	**Mean (USD)**	**Sum**
Year	2014	2480	3940	13.63	1.27	6.36	1,599,282
	2015	1137	1376	15.45	.83	6.56	1,784,051
	2016	1262	1717	16.53	.77	2.09	1,769,902
	2017	1264	1552	16.33	.76	2.60	1,791,857
	2018	1217	1458	12.81	.64	12.53	1,734,966
	2019	578	1107	16.61	.63	20.92	1,480,740
	2020	748	1098	15.51	.44	24.54	1,471,526
	2021	891	930	15.03	.59	19.53	1,434,482
	Total/average	9577	13178	15.29	.77	11.23	1,475,000 ^g^

^a^CIBE: conflict intensity best estimate (N)

^b^CIHE: conflict intensity high estimate (N)

^c^GAM: global acute malnutrition (% of population 6–59 months)

^d^U5CMR: under-five crude mortality rate (Rate per 10,000/day)

^e^Based on sorghum prices (USD for a 3.5 kg unit of white sorghum)

^f^Based on OCHA 2020 Population estimation (total number of displaced population)

^g^average IDPs per year

**Fig. 3:**
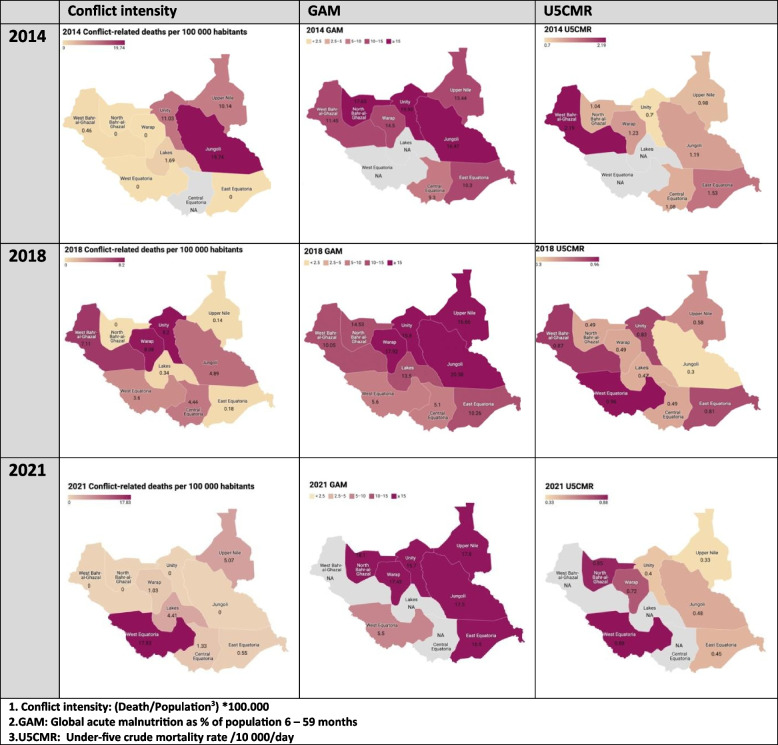
Trend of conflict intensity, GAM, and U5CMR2 in 2014, 2018 and 2021

The intensity of conflicts, as measured by death rate per 100.000 per year, was highest in 2014 (3.78) and showed a general decreasing trend, reaching its lowest in 2020 (1.21). Unity, Jonglei, and Upper Nile were the most affected regions, while Eastern Equatoria and Western el Ghazal reported fewer deaths. Notably, Northern Bahr el Ghazal reported no deaths throughout the study, see Fig. 3.

Regarding malnutrition, the data revealed a consistently high prevalence of global acute malnutrition (GAM) among children under five, with the mean GAM rate across the years being 15.29. The northern and eastern states, including Warrap, Unity, Jonglei, Upper Nile, and Northern Bahr el Ghazal, exhibited very high GAM rates, see Table 1 and Fig. 3.

The under-five crude mortality rate (U5CMR) in South Sudan showed a declining trend, from 1.27 in 2014 to 0.59 in 2021. The highest U5CMR rates were observed in Eastern Equatoria, Western Bahr el Ghazal, and Western Equatoria, which contrastingly had among the lowest numbers of conflict-related deaths. Northern Bahr el Ghazal, with no reported conflict-related deaths, recorded the lowest mean U5CMR (0.54) over the eight years, see Table 1. Figure 3 illustrates the conflict intensity, GAM and U5CMR in year 2014, 2018 and 2021.

Geographical visualization of the conflict intensity, global acute malnutrition, and under-five crude mortality rate in South Sudan between 2014–2021, presented in three cut-off points (2014, 2018, 2021).

**Fig. 4:**
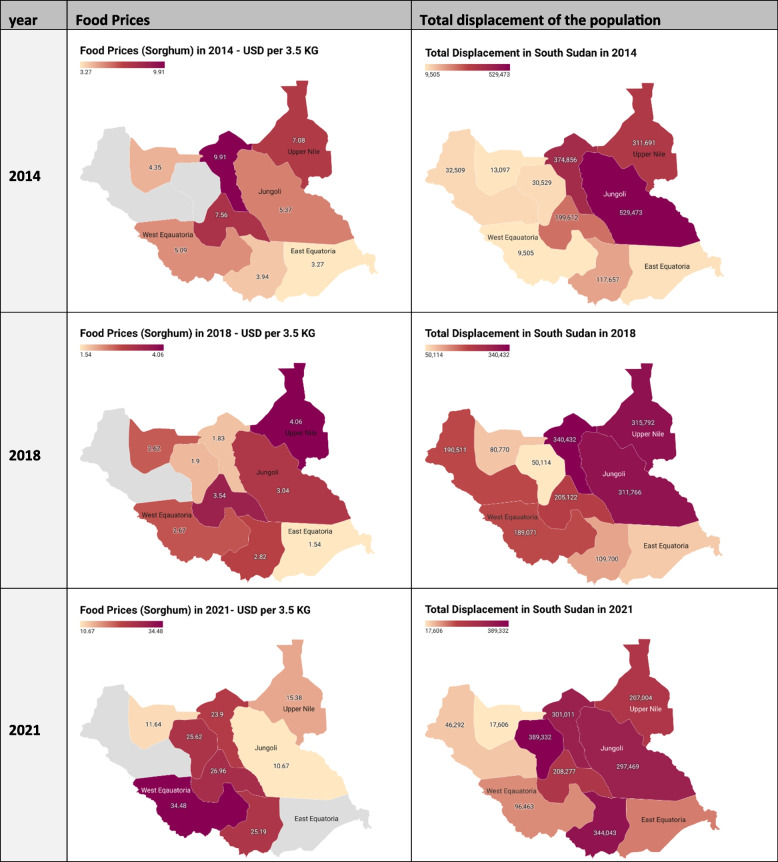
Displacement and food prices per state in South Sudan (2014, 2018, 2021)

The average sorghum price, valued in USD for a 3.5 kg unit, had an overall upward trend from $6.36 in 2014 to $19.53 in 2021, with a peak at $24.54 in 2020. Concurrently, the internal displacement figures exhibited a fluctuating trend, with the total number of internally displaced individuals starting at 1,599,282 in 2014, reaching a high of 1,784,051 in 2015, and eventually decreasing to 1,434,482 by 2021. Figure 4 represents the average food prices and internal displacement per state in year 2014, 2018 and 2021.

### Correlation analysis between the research variables

Table 2 presents the correlations between GAM, U5CMR, conflict intensity (best and high estimates), food prices (Sorghum) and displacement. GAM showed a weak positive significant correlation with the high estimate of conflict intensity (CI = 0.013–0.090, *r* = 0.158, *p* = 0.047), while no significant difference was noticed with the best estimate of the conflict intensity (*p* = 0.553). Similarly, food prices, as measured by the cost of Sorghum, exhibited a moderate positive correlation with GAM (CI = 0.007–0.186, *r* = 0.171, *p* = 0.048), indicating that rising food prices are associated with an increase in global acute malnutrition rate among children. Displacement had a positive correlation with GAM (CI = 0.010–0.297, *r* = 0.293, *p* = 0.016).

**Table 2:** Correlation between GAM, U5CMR and the research variables

	**Global acute malnutrition as % of population 6–59 months**			**Under 5 crude mortality rate / 10 000 / day**		
	**CI**^**1**^	**p**	**Pearson’s Correlation**	**CI**^**1**^	**p**	**Pearson’s Correlation**
**Conflict intensity best estimate**	−0.009–0.020	.553	0.203	−0.003 – 0.000	0.065	−0.030
**Conflict intensity high estimate**	**0.013–0.090**	.047*	**0.158**	**0.090 – 0.200**	0.043*	**0.010**
**Food prices (Sorghum)**	**0.007–0.186**	.048*	**0.171**	−0.015 – 0.002	0.134	−0.188
**Internal Displacement**	**0.010–0.297**	.016*	**0.293**	0.000–0.000	0.652	−0.002

^*^ Significant correlation

^1^confidence interval

In contrast, the under-5 mortality rate (U5CMR) displayed different degrees of association with the studied variables. Conflict intensity, with high estimate, showed a very weak positive correlation with U5CMR (CI = 0.090–0.200, *r* = 0.010, *p* = 0.043), indicating a marginal impact on child mortality rates. Interestingly, the correlation between U5CMR and food prices (Sorghum) was negative (*r* = −0.188, *p* = 0.134), though not statistically significant, suggesting that the relationship between food prices and U5CMR might be influenced by other mitigating factors. Displacement showed an almost negligible correlation with U5CMR (*r* = −0.002, *p* = 0.652).

**Fig. 5:**
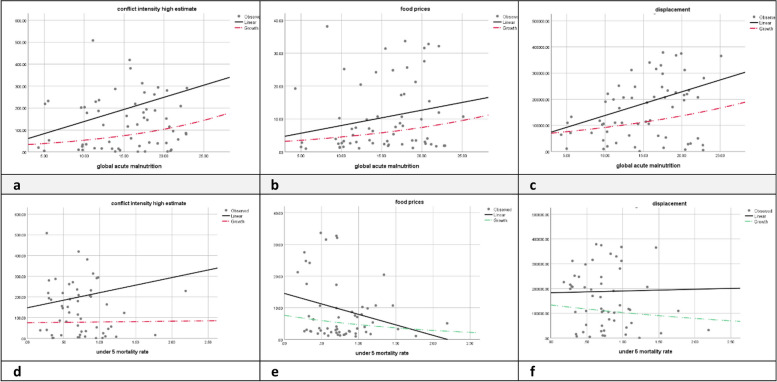
**a**-**f** The correlation between GAM, U5CMR with conflict intensity, food prices, and displacement in South Sudan between the year 2014–2021

The scatter plots illustrate that higher conflict intensity correlates with increased global acute malnutrition (GAM), suggesting conflicts exacerbate malnutrition. Rising food prices also relate positively to GAM, indicating economic factors contribute to nutritional deficits. Displacement shows a pronounced positive correlation with GAM, highlighting it as a significant factor in malnutrition, see Fig. 5.

Conversely, under-5 mortality rate (U5CMR) exhibits a mild positive correlation with conflict intensity, indicating some impact, but a negative correlation with food prices, suggesting more complex interactions. Displacement appears to have a minimal direct relationship with U5CMR, pointing to other mediating influencess, see Fig. 5.

## Discussion

The study highlights the intricate and complex relationship between armed conflict and child health. Through a panel data analysis, we sought to quantify the direct effects of conflict-related incidents on these health metrics. Our results showed significant positive correlation between high estimate of conflict intensity, food prices, and displacement with GAM, however, the best estimate of conflict intensity didn’t show any significant difference with the research variables.

The conflict stifled food production which led to a historical increase in food prices and hyperinflation [37]. In 2014 one kilogram of Sorghum, which is the main nutritional source for the South Sudanese population, costs approximately 300% more compared to previous years, which is represented in our result by the significant correlation between food prices and GAM [37]. In 2021 there has been a sharp increase in Sorghum prices which can be linked to inflation caused by the COVID-19 pandemic [38]. Moreover, the conflict has displaced 1.8 million people internally, and 1.3 million people to the surrounding countries, and those were the most in need for humanitarian aid and affected by food insecurity [39], represented by significant correlation between displacement and GAM in our results. This explains the hypothetical framework of this study as food prices and displacement are interlinked and plays explaining factor of how the conflict intensity affect child health [40].

Further examination revealed that states with higher numbers of conflict-related deaths also exhibited elevated GAM rates, particularly in the northern regions of South Sudan. Interestingly, U5CMR did not show a similar pattern, with rates decreasing consistently from 2014 to 2021 across the states, regardless of conflict intensity. This was also presented statistically with weak correlation between U5CMR and conflict intensity, and negative correlation between food prices/displacement and U5CMR. This unexpected trend underscores the complexity of conflict's impact on child health, suggesting that other factors, possibly including humanitarian interventions and access to health services, may play significant roles [13, 15].

A previous study in Nigeria which used UCDP to investigate the nutritional outcome of children exposed to conflict has found that armed conflict is significantly associated with long-term nutritional outcomes for children. Those outcomes have great implication on the children’s health and future cognitive performance [5]. However, another systematic review published in 2022 suggests a significant and negative association between conflict and child malnutrition, diverging from our study's findings. This discrepancy highlights the complexity of collecting data from a conflict zone, and the challenges of assessing conflict's impact on child health and the influence of methodological and data limitations. The review also points out potential biases due to excluding severe conflict zones and not accounting for displaced or migrated children's origins, which could skew the true effect of conflict on child health [41].

The three states with the highest intensity of armed conflicts (Unity, Jonglei and Upper Nile) also had the highest number of SMART surveys conducted by humanitarian organizations. A study from Democratic Republic of the Congo [42] suggest that armed conflict alone is a bad indicator for prediction for child health. This study and the study conducted in Congo shows that children living in conflict areas are more likely to get humanitarian and health assistance. This could send a signal that humanitarian organizations should avoid focusing exclusively on regions with active fighting, as other parts of the country with less conflict can also suffer severe impacts and risk being forgotten.

The civil war in South Sudan represents an example how conflict can affect health outcome of a population. This study summarizes the challenges of conducting research in conflict context, where multi variables can contribute to the outcome, and the data can be difficult to collect and verify, which results in gaps in our understanding of the true impact of conflict on health. These gaps underscore the critical need for innovative, conflict-sensitive research methodologies that can navigate the complexities of war-torn regions. Future studies should aim to incorporate more granular data and analytical techniques, to enhance the precision of estimates and provide more definitive insights into the relationship between armed conflict and child health.

### Limitations

Our results should be interpreted taking into consideration some limitations. The UCDP database was chosen as the main provider of data during conflict and produces high quality data, which is systematically collected and organized, and all the data included is verified and linked to a geographical location. However, the total number of death registered at UCDP in South Sudan between 2014–2021 was on the high estimate a little over 13 thousand which is relatively small compared to the estimation of the casualties during the same period which has been estimated to be as high as 190 thousand deaths [14, 36]. The UCDP database, while registering fewer conflict-related deaths compared to other sources, is deemed the most accurate, highlighting the challenges of relying on estimations from less precise sources. Importantly, the lack of statistical significance in our findings does not undermine the adverse effects of armed conflict on child health and mortality. This discrepancy underscores the complexities in quantifying conflict's impact on health outcomes and the necessity for ongoing research and methodological refinement in this domain.

The SMART survey database had several limitations as well, such as an uneven distribution of surveys, missing values, and data collection during different periods of the year, which can influence the results of the surveys and were not accounted for in the study.

## Conclusion

The effect of conflict on children’s health outcome is complicated and multifactorial. The high estimate of conflict intensity from UCDP showed significant correlation with the health outcome, while best estimate did not have significant correlation, this could be due to limited health data, underreporting of conflict-related deaths and a small sample size. The study suggests that other factors such as food prices and internal displacement play an additional contributing factor which amplifying the effect of conflict intensity on child health. It underscores the challenge of data scarcity in researching health determinants in South Sudan.

## Data Availability

No datasets were generated or analysed during the current study.

## Appendix

**Table:**

**Number of deaths for high estimate conflict intensity (N of deaths/ state population *100,000)**									
	**2014**	**2015**	**2016**	**2017**	**2018**	**2019**	**2020**	**2021**	**total**
**Central Equatoria**	10 (0.66)	17 (1.13)	508 (33.69)	236 (15.6)	219 (14.52)	219 (14.52)	178 (11.8)	35 (2.32)	1422 (94.3)
**East Equatoria**	.	2 (0.18)	16 (1.46)	164 (14.9)	35 (3.19)	46 (4.19)	N/A	27 (2.46)	290 (26.41)
**Jonglei**	1009 (50.90)	1 (0.05)	271 (13.67)	294 (14.8	280 (14.13)	152 (7.67)	687 (34.66)	156 (7.87)	2850 (143.78)
**Lakes**	25 (2.12)	187 (15.84)	62 (5.25)	262 (22.2)	15 (1.27)	123 (10.42)	116 (9.83)	106 (8.98)	896 (75.92)
**North Bahr-al-Ghazal**	97 (10.63)	2 (0.22)	2 (0.22)	N/A	N/A	N/A	5 (0.55)	N/A	106 (11.62)
**Unity**	1462 (100.3)	701 (28.55)	57 (26.55)	189 (10.4)	381 (11.4)	287 (3.74)	39 (0.91)	N/A	3116 (199.68)
**Upper Nile**	1100 (75.36)	313 (21.44)	291 (19.94)	115 (7.88)	125 (8.56)	41 (2.81)	10 (0.69)	194 (13.29)	2189 (149.96)
**Warap**	8 (0.63)	95 (7.52)	86 (6.81)	81 (6.42)	146 (11.56)	209 (16.55)	43 (3.41)	180 (14.26)	848 (67.17)
**West Bahr-al-Ghazal**	229 (35.41)	25 (3.87)	419 (64.79)	202 (31.23)	204 (31.54)	15 (2.32)	N/A	N/A	1094 (169.16)
**West Equatoria**	.	33 (3.61)	5 (0.55)	9 (0.98)	53 (5.8)	15 (1.64)	20 (2.19)	232 (25.37)	367 (40.14)
**Mean Global Acute Malnutrition 2014–2021—Percentage**									
	**2014**	**2015**	**2016**	**2017**	**2018**	**2019**	**2020**	**2021**	**Mean GAM**
**Central Equatoria**	9.30	11.30	11.10	11.86	5.10	16.40	10.35	.	10.77
**East Equatoria**	10.30	13.90	12.20	15.32	10.26	13.15		16.93	13.15
**Jonglei**	16.47	16.73	17.55	19.26	20.38	20.30	22.10	17.50	18.79
**Lakes**	.	11.80	15.88	18.80	13.50	12.40	15.47		14.64
**North Bahr-al-Ghazal**	.	17.63	20.45	.	.	.	20.82	.	19.63
**Unity**	19.93	25.13	21.78	18.88	15.80	13.90	19.21	.	19.23
**Upper Nile**	13.44	17.28	22.87	20.68	16.66	20.30	20.92	17.80	18.74
**Warap**	14.50	21.15	22.75	22.78	17.92	22.10	18.16	17.43	19.60
**West Bahr-al-Ghazal**	11.45	9.76	15.70	9.63	10.05	14.30	8.24	.	11.30
**West Equatoria**	.	9.80	5.00	9.80	5.60	.	4.28	5.50	6.66
**Mean Under-Five Crude Mortality Rate (U5CMR) Result 2014–2021 (death per 10.000 child/day)**									
	**2014**	**2015**	**2016**	**2017**	**2018**	**2019**	**2020**	**2021**	**Mean**
**Central Equatoria**	1.08	0.73	0.27	0.69	0.49	0.29	.	.	0.59
**East Equatoria**	1.53	0.35	1.77	1.10	0.81	1.12	.	.	1.11
**Jonglei**	1.19	0.95	0.63	0.98	0.30	0.56	.	0.48	0.73
**Lakes**	.	0.33	0.70	0.58	0.47	1.34	.	.	0.68
**North Bahr-al-Ghazal**	.	1.04	0.36	.	.	.	0.70	.	0.70
**Unity**	0.70	1.46	1.16	0.59	0.83	0.39	0.18	.	0.76
**Upper Nile**	0.98	0.91	0.96	0.70	0.58	0.27	.	0.30	0.67
**Warap**	1.23	0.74	0.45	0.54	0.49	0.71	.	0.70	0.69
**West Bahr-al-Ghazal**	2.19	1.05	0.71	0.88	0.87	0.34	.	.	1.01
**West Equatoria**	.	0.75	0.72	0.80	0.96	.	.	0.88	0.82
**Sorghum prices in South Sudan per state (in USD, 3.5 KG unit) 2014–2021**									
	**2014**	**2015**	**2016**	**2017**	**2018**	**2019**	**2020**	**2021**	**Mean**
**Central Equatoria**	3.94	6.98	2.61	2.99	2.82	24.80	25.16	25.19	11.81
**East Equatoria**	3.27	2.63	1.09	2.36	1.54	9.82	.	.	3.45
**Jonglei**	5.37	7.57	2.30	3.46	3.04	31.56	11.95	10.67	9.49
**Lakes**	7.56	7.35	2.16	3.47	3.54	20.45	31.38	26.96	12.86
**North Bahr-al-Ghazal**	4.35	7.89	2.51	.	.	.	32.76	.	11.88
**Unity**	9.91	10.71	1.70	1.19	1.83	6.33	21.24	.	7.56
**Upper Nile**	7.08	8.70	1.87	4.06	62.71	27.53	15.38	17.56	18.11
**Warap**	10.72	2.31	1.86	1.90	33.66	32.11	25.62	17.27	15.68
**West Bahr-al-Ghazal**	5.09	10.24	3.27	2.49	2.67	24.19	38.12	.	12.30
**West Equatoria**	.	1.26	1.49	1.45	0.97	11.51	19.24	.	5.99
**Internal displacement in South Sudan per state 2014–2021**									
	**2014**	**2015**	**2016**	**2017**	**2018**	**2019**	**2020**	**2021**	**Total**
**Central Equatoria**	117657	110219	198977	165833	109700	208853	220847	344043	1476129
**East Equatoria**	19787	16085	109857	114125	75622	61225	.	60076	456777
**Jonglei**	529473	613973	378821	367912	311766	196055	207154	297469	2902623
**Lakes**	199612	251712	118854	170134	205122	206455	187241	208277	1547407
**North Bahr-al-Ghazal**	13097	14157	7101	.	.	.	28123	.	62478
**Unity**	374856	365511	534689	540085	340432	247614	225963	.	2629150
**Upper Nile**	311691	329691	280723	219645	315792	215888	233814	207004	2114248
**Warap**	600	30529	24229	5272	53462	50114	233198	246697	644101
**West Bahr-al-Ghazal**	32509	42669	105846	102821	190511	105465	71159	.	650980
**West Equatoria**	.	9505	10805	106030	132559	189071	64027	70916	582913
